# Child abuse predicts adult PTSD symptoms among individuals diagnosed with intellectual disabilities

**DOI:** 10.3389/fpsyg.2015.01600

**Published:** 2015-10-19

**Authors:** Claudia Catani, Iris M. Sossalla

**Affiliations:** Department of Psychology, Bielefeld University, Bielefeld, Germany

**Keywords:** child abuse, victimization, intellectual disabilities, trauma, PTSD

## Abstract

Prior research has shown that people with intellectual disabilities (ID) are more likely to experience child abuse as well as other forms of traumatic or negative events later in life compared to the general population. Little is known however, about the association of these experiences with adult mental health in intellectually disabled individuals. The present study aimed to assess whether child abuse in families and institutions as well as other types of adverse life events, were associated with current posttraumatic stress disorder (PTSD) and depression symptoms in individuals with ID. We conducted clinical interviews which included standardized self-report measures for childhood abuse, PTSD, and depression in an unselected sample of 56 persons with a medical diagnosis of ID who were attending a specialized welfare center. The frequency of traumatic experiences was very high, with physical and emotional child abuse being the most common trauma types. 87% of the persons reported at least one aversive experience on the family violence spectrum, and 50% of the sample reported a violent physical attack later in adulthood. 25% were diagnosed with PTSD and almost 27% had a critical score on the depression scale. Physical and emotional child abuse was positively correlated with the amount of institutional violence and the number of general traumatic events, whereas childhood sexual abuse was related to the experience of intimate partner violence in adult life. A linear regression model revealed child abuse in the family to be the only significant independent predictor of PTSD symptom severity. The current findings underscore the central role of child maltreatment in the increased risk of further victimization and in the development of mental health problems in adulthood in intellectually disabled individuals. Our data have important clinical implications and demonstrate the need for targeted prevention and intervention programs that are tailored to the specific needs of children and adults with intellectual disability.

## Introduction

There is growing evidence that individuals with disabilities constitute a particularly vulnerable group with regard to likelihood of abusive experiences during childhood as well as later in life. Two recent meta-analyses have reported alarming prevalence rates of violence against disabled adults (Hughes et al., 2012) and children (Jones et al., 2012). The increased risk of having experienced violence in the last year in adults with disabilities compared to the healthy population is approximately 50% (Hughes et al., 2012). Children with disabilities are more than three times more likely to have experienced violence in their lives than non-disabled children. More specifically, children with mental or intellectual impairments seem to have a higher prevalence and risk of violence than do children with other types of disability (Jones et al., 2012). This finding is consistent with studies showing that adults with intellectual disabilities (ID) have the highest population rates of violence compared with both the general population and persons with other disability types (Lin et al., 2009; Rand and Harrell, 2009).

Even though there is a widespread view that disability increases the likelihood for children to be abused by parents or other caretakers (Spencer et al., 2005), there is still a paucity of research examining specific types of child abuse (e.g., neglect vs. physical violence vs. emotional violence) and their association with specific types of disability (e.g., physical vs. intellectual). Many studies are methodologically weak and rely on medical or school records or information from caretakers or social workers rather than subjective information provided directly by the disabled individuals. A population-based study by Sullivan and Knutson (2000a) assessed the prevalence of abuse and neglect among children with and without disabilities by relating specific types of disabilities to specific types of abuse. The analysis of school records and official databases revealed a 31% maltreatment rate (including sexual, physical, emotional abuse, and neglect) among children with disabilities compared to a 9% rate in non-disabled children. Neglect was the predominant form of maltreatment, followed by physical abuse, emotional abuse and sexual abuse. The maltreatment risk was particularly high for children with mental retardation and learning disabilities, when compared to children with physical impairments.

A number of studies show that violence in the lives of people with disabilities usually does not end in childhood but rather continues throughout the life span with intimate partner violence (IPV) being a very frequent phenomenon. Women with disabilities (physical and intellectual) are more likely to be abused by their partners than women without disabilities, and for longer periods of time (Nosek et al., 2001; Smith and Strauser, 2008; Hughes et al., 2011; Scherer et al., 2014). In addition to experiencing subtle forms of abuse that exploit aspects of disability, such as withholding medication or denying needed supports (Lund, 2011), they are also more often victims of severe forms of physical partner violence, including being kicked or punched (Brownridge, 2006). Even though women in general are substantially more likely to experience severe domestic or dating violence (Black et al., 2011), men with disabilities are also vulnerable to victimization by their partners. Two population-based studies have examined IPV against men with disabilities and showed a greater prevalence of lifetime and past-year IPV victimization amongst disabled men compared to non-disabled men (Cohen et al., 2006; Mitra and Mouradian, 2014). The high rates of child abuse in disabled individuals, together with the resulting psychological problems, can be assumed to be potential contributing factors to the frequent experience of IPV. In fact, research with non-disabled samples has repeatedly shown that abusive experiences in early childhood increase the likelihood of being re-victimized by a spouse or dating partner in adult life (Desai et al., 2002; Bensley et al., 2003).

Most of the existing studies on traumatic experiences in the lives of disabled individuals have focused on child abuse or domestic violence whereas only limited research has addressed other types of traumatic events or life adversities in this specific population. The National Crime Victimization Survey (Rand and Harrell, 2009) investigated the frequency of non-fatal violent crimes (e.g., rape, robbery, aggravated assault) and property crimes (e.g., household burglary, property theft) in persons aged 12 or older with physical or cognitive disabilities. Results showed that persons with disabilities were 1.5 times more likely to experience a violent crime and two times more likely to be sexually assaulted than persons without disabilities. Other evidence shows that most of the violence takes place within the social environment of the victim and that individuals with disabilities are most often victimized by someone close to them or by service providers (Sullivan and Knutson, 2000b; Reiter et al., 2007). It has been suggested, that disabled persons often become victims because they are considered to be vulnerable, helpless and not able to report the crime (Sobsey, 1994). In addition, an early study has shown that, even when crimes are reported by individuals with disabilities, there are lower rates of police follow-up, prosecution and convictions as well as lighter sentences when convictions do occur (Sobsey and Doe, 1991).

In sum, despite a number of gaps in the research, there is evidence that individuals with disabilities have a higher probability of experiencing adverse life experiences, particularly child abuse committed by family members or other caretakers. However, a critical question that remains unanswered is whether disabled children and adults are also vulnerable to the disruptive effects of trauma on mental health and psychosocial functioning. Amongst non-disabled individuals, the long-term detrimental consequences of childhood abuse and other early traumatic experiences are well documented. Childhood trauma has been repeatedly associated with poor mental health in adults, especially with a higher risk for depression (Heim et al., 2008; Nanni et al., 2012) and posttraumatic stress disorder (PTSD; Koenen et al., 2007; Sugaya et al., 2012). An increased risk of physical problems later in life has also been demonstrated (Knudsen et al., 2006; Tischler et al., 2006; Shonkoff et al., 2012). However, little systematic research has explored whether the consequences of violence against individuals with disabilities are quantitatively or qualitatively different from non-disabled persons. Individuals with ID might be more likely to develop psychological disorders in the aftermath of exposure to trauma. In addition to reduced cognitive coping mechanisms, external factors such as an increased dependency on others, early institutionalization, limited social support (Martorell et al., 2009) or being surrounded by a high risk peer network (Kepper et al., 2014) might play a role in increasing the vulnerability of this group.

However, the empirical evidence linking childhood trauma and mental ill-health and specifically PTSD in individuals with ID is very limited even though studies from the general population suggest an association between PTSD risk and intelligence. For instance, a prospective study following children over a time interval of 11 years found that higher levels of intelligence were associated with a decreased conditional risk for PTSD (Breslau et al., 2006). Consistent with this, research with combat veterans showed that lower levels of intelligence were linked to a greater risk of developing PTSD symptoms (Macklin et al., 1998). Different results were yielded by a very recent study with Israeli civilians with ID (Berger et al., 2015) exposed to war violence (missile attacks). Similar to the research findings for non-disabled people, war exposure in this group was related to Posttraumatic symptoms and functioning problems, but the rate of mental health impairments was low compared to the general exposed Israeli population. However, study results might be questionable since they were exclusively based on an assessment of the professional caretaker.

Taken together, evidence from studies with disabled and non-disabled individuals suggest that survivors of child abuse with ID have an elevated risk for developing PTSD compared to non-abused samples. However, it remains unclear whether this risk is different from that of non-disabled individuals who experienced child abuse (Mevissen and de Jongh, 2010). In addition, very little is known about potential risk factors associated with PTSD in adults with ID. Based on studies with the general population and high-risk populations without disabilities, it can be assumed that the amount of traumatic experiences of different types (child abuse, IPV) plays a critical role for the development of PTSD (Gilbert et al., 2009; Catani and Ruck, 2012). Recent evidence has proven that institutional child abuse, too, has a negative impact on the mental health of adult survivors (Fitzpatrick et al., 2010) and is related to a diagnosis of PTSD in adult life (Lueger-Schuster et al., 2014). Yet, there is no research addressing the relationship between institutional child abuse and PTSD in intellectually disabled people.

The current study aimed to assess child abuse and later traumatic life events as well as current PTSD and depressive symptoms in an unselected sample of individuals with ID. We hypothesized that the amount of different types of violent and adverse experiences including childhood maltreatment in families or institutions and IPV, would be associated with PTSD symptom severity. In addition, we assumed that participants with many experiences of child abuse would report a greater severity of current depression symptoms compared to individuals without or with less abuse.

## Materials and Methods

### Sample

The sample consisted of 56 persons who were attending a welfare center that specialized in the care and support of people with ID. Because of concerns related to participants’ burden and due to logistical reasons, a formal IQ testing could not be carried out. Instead, we used the presence of a formal diagnosis of intellectual disability (ID) in the participant’s medical records as primary inclusion criterion. In addition, participants had to be aged over 18 to be eligible for this study. Exclusion criteria were the presence of cognitive problems that were too severe to allow for an adequate understanding of interview contents, current psychotic disorder or acute suicide ideation. We invited the social workers working at the center to inform all of their clients who met the inclusion criteria about the study. We also instructed the social workers to document the number of persons they had informed and the reason for failing to inform some of their cases. Since some social workers opted out of the study, we did not obtain any information for 30% of the approximately 140 users of the welfare center. Another 20 persons were not informed about the study because their social workers had the impression they were too stressed or burdened with daily hassles to participate in the interview. In total, 88 persons with ID attending the center were informed about the study and asked to participate. Out of those, 17 refused to take part and 71 persons opted in. Reasons for not participating were mainly “no interest” or “being afraid to talk to strangers.” We were able to interview 56 people within the available time period. Table 1 provides an overview of the socio-demographic characteristics of the final group of participants.

**TABLE 1:** **Sociodemographic and other characteristics of participants (*N* = 56)**.

Gender, % male (*n*)	54(30)
Age, *M* (SD)	41.5 (13, 3)
Nationality, % German (*n*)	96.4 (54)
Family status, % (*n*)	
Single	46.4 (26)
Not married, but stable partner	32.1 (18)
Married	8.9 (5)
Other	12.5 (7)
Degree of education, % (*n*)	
No degree	35.2 (19)
Certification from a special-needs school	37.0 (20)
Certificate of secondary education	18.5 (10)
Other (higher) degrees	8.9 (5)
Unknown	3.6 (2)
Current employment status, % (*n*)	
Working in protected environments (specifically for people with ID)	73.2 (41)
Unemployed	5.4 (3)
Retired	7.1 (4)
Other	14.3 (8)
Part of childhood spent in a child care center, % yes (*n*)	32.1 (18)

### Procedure

Participants who volunteered to take part in the study arranged individual appointments through their personal social worker who accompanied them to the interview. All interviews took place in a quiet and private room at the welfare center and were conducted by one of the authors. Participants were invited to choose whether they wanted their social worker to be present during the interview or not. All but one (*n* = 55) participants wanted their social workers to stay. Before the interview started, aim and content of the study, procedure, risks, the right to withdraw and confidentiality were explained and written consent was obtained. The clinical examination was conducted as a standardized interview. We also included self-report questionnaires that were all administered as a face-to-face interview in order to enhance clarity and to adapt questions to the cognitive capabilities of the respective participant, if needed. Interviews lasted on average 60 min. The study was approved by the Ethical Review Board of the University of Bielefeld.

### Instruments

#### Sociodemographic and Clinical Information

The first part of the interview comprised of a series of questions about individual sociodemographic characteristics and clinical information related to the type of disability, medical and psychiatric diagnoses, current or past psychiatric or psychological treatments, intake of medication, acts of self-injury, and suicide attempts.

#### Adverse Childhood Experiences and Intimate Partner Violence

For the assessment of adverse childhood experiences we created a 22—item checklist by selecting items from a questionnaire that had previously been used with children in various cultural contexts (Catani et al., 2008). Following discussions with clinical experts and social workers involved in the daily work with persons with ID, the most suitable items for this specific group of people were chosen and wording was adapted if needed. The final checklist consists of five items asking about physical abuse, e.g., “Have you been hit with an object?”, three items addressing sexual abuse, e.g., “Have you been forced to have sexual intercourse with a family member (a teacher/child care worker)?”, four items asking about emotional abuse, e.g., “Have you been told that you are stupid or ugly?”, and four items addressing neglect, e.g., “Were you not given enough to eat or drink?”. Two additional items ask about witnessing violence, e.g., “Have you seen your mother being hit at home?”. For each item, participants were asked whether they had experienced the specific event type as a child either in their family environment or in a children’s home or daycare institution or school. Two further questions explored whether the participants had injuries (e.g., bruises, broken bones) or needed medical treatment as a consequence of the experienced violence. An index of the severity of childhood abuse was established by counting the numbers of event types reported by the participants, separately for incidences that had happened at home/in the family (*family abuse*) and those that took place in a particular institutional setting (*institutional abuse*).

At the end of the questionnaire, participants were asked whether they had ever been beaten by an intimate partner.

#### Posttraumatic Stress Disorder

DSM-IV diagnosis and severity of PTSD were assessed using the German version of the Posttraumatic Stress Diagnostic Scale (PDS; Steil and Ehlers, 2000) carried out in interview form. The PDS is a DSM-IV-based self-report measure and includes a checklist of 12 traumatic events as well as 17 symptom items for the assessment of past-month PTSD symptoms. The number of experienced or witnessed traumatic events is determined by counting every positive response to the event checklist. Frequency of each of the 17 symptoms items is coded on a 4-point Likert-type scale ranging from 0 (never or only once in the past month) to 3 (almost daily in the past month). The symptom severity score for each symptom cluster is obtained by summing scores of the respective items for cluster B, C, and D. A sum score for overall PTSD severity score is provided by the total score of all symptom items (range: 0–51). Symptom severity was rated based on the cut-offs provided in the original manual (Foa, 1995). A diagnosis of PTSD was made on the basis of DSM-IV criteria. The PDS has previously been adapted and used for the assessment of PTSD in individuals with ID (Mitchell et al., 2006; Brackenridge, 2010).

#### Depression and Substance Use

We employed the German Version (Glaesmer et al., 2014) of the 15-item depression section (DHSCL) of the Hopkins Symptom Checklist (HSCL) to measure current symptoms of depression. The DHSCL uses a 4-point Likert scale ranging from 0 (“not at all”) to 3 (“extremely”). The severity of depression symptom was calculated on the basis of the total scale mean score. The presence of clinically relevant depression symptoms was derived using the internationally accepted cutoff score of 1.75 (Derogatis et al., 1974; Mollica et al., 1987).

To assess participants’ level of alcohol use, we employed four questions asking (1) whether the participants drank alcohol, (2) how often they drank alcohol, (3) whether they had tried and failed to stop drinking in the past 12 months, and (4) whether a relative, friend or health worker had been concerned about their drinking. In addition, participants were asked whether they consume any other substance.

### Data Analysis

All statistical analyses were carried out using STATISTICA, Version 8.0 (StatSoft, 2007). Associations between different types of adverse and traumatic experiences were explored using Spearman rank correlations. In order to identify risk factors for severity of PTSD symptoms, we conducted a linear regression analysis on the PDS sum score. The model was adjusted for gender and age. We entered exposure to IPV, the number of traumatic events, the amount of family violence, and the number of experiences related to institutional violence as predictor variables in the model. Spearman’s rho was calculated for continuous predictor variables and point-biserial correlations for dichotomous predictor variables. The linear regression model was adjusted for gender and age. To compare clinical outcomes between subgroups of the sample, the Student’s *t*-test for unpaired samples was used whereas chi-square tests were employed for categorical variables.

## Results

### Childhood Abuse

**FIGURE 1:**
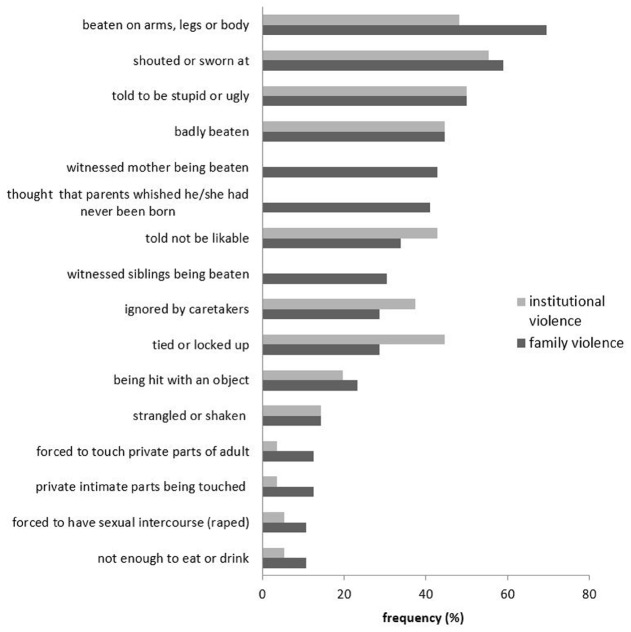
**Frequency (%) of types of abusive experiences reported most often by the participants (separately for events experienced at home and in children’s homes, school, or daycare institutions)**.

Forty-nine (87.5%) of the participants reported at least one type of abusive event as a child in their family and still 28 (50%) had experienced four or more different types. 76.8% of the sample reported at least one form of emotional abuse, 73.2% reported physical abuse, 39.3% reported experiences of neglect and 12.5% had experienced at least one incidence of sexual violence in their families. 48.2% of the participants had witnessed their mother or siblings being beaten at home. On average, participants reported 5.2 (SD = 4.2) out of 18 possible different types of child abuse experiences happening in their families. Figure 1 illustrates the frequency of the most common types of events described by the participants.

The majority of the sample also reported a variety of child abuse experiences that had happened in a particular institution (see Figure 1), e.g., in a children’s home, a daycare unit or at school. 45 participants (80.4%) had experienced at least one form of institutional abuse and 55.4% reported four or more different types. 69.6% of the sample reported at least one type of emotional abuse, 76.8% reported physical abuse, 42.9% reported experiences of neglect and 5.3% had experienced at least one incidence of sexual violence in a child care or educational institution. On average, participants reported four (SD = 3.1) different types of institutional abuse experienced as a child.

Regarding the consequences of these abusive experiences, 42.9% of the participants had suffered at least one injury because of the violent experiences at home or in institutions and 17.9% of them had needed medical treatment on at least one of those occasions.

### Trauma Exposure

Fifty-two participants (92.9%) reported at least one traumatic event on the PDS event checklist. On average, they reported 3.6 (SD = 2.1) different events. The most frequently mentioned traumatic experiences were non-sexual assaults by a family member or someone known (53.6%, *n* = 30), non-sexual assaults by strangers (50%, *n* = 28), serious accidents (50%, *n* = 28), and a life-threatening illness (48.2%, *n* = 27). 14.3% (*n* = 8) of the sample reported a sexual assault by a family member or someone known. 41.1% (*n* = 26) stated they had experienced something described as “other traumatic events.” Examples for this category were “*Surgery in my childhood*,” “*stillbirth*,” “*sudden death of my husband*.”

The question “*Have you ever been beaten by an intimate partner*” was answered positively by 10.7% of the male participants (*n* = 3) and 45.8% (*n* = 12) of the females.

### Association Between Different Types of Adverse and Traumatic Experience

As shown in Table 2, there were a number of significant correlations between the different types of adverse experiences in childhood and adult life. Physical and emotional abuse in the family were significantly associated with every other form of abuse and trauma except for neglect. Institutional violence significantly correlated with abusive experiences in the family, i.e., physical and emotional abuse and the witnessing of family violence.

**TABLE 2:** **Spearman rank correlations between different types of childhood abuse and traumatic experiences**.

	**Physical abuse (family)**	**Emotional abuse (family)**	**Neglect (family)**	**Sexual abuse (family)**	**Witnessing family Violence**	**Institutional Violence (total)**	**Intimate Partner violence (y/n)**	**Number of traumatic events**
Physical abuse (family)	–	0.66**	0.01	0.36**	0.39**	0.49**	0.07	0.50**
Emotional abuse (family)	–	–	–0.07	0.35**	0.56**	0.78**	–0.02	0.54**
Neglect (family)	–	–	–	0.06	–0.11	–0.06	0.14	0.04
Sexual abuse (family)	–	–	–	–	0.18	0.14	0.35**	0.24^+^
Witnessing family violence	–	–	–	–	–	0.43**	–0.01	0.20
Institutional violence (total)	–	–	–	–	–	–	–0.19	0.47**

**Indicate correlations significant on p < 0.01. ^+^Indicates a statistical trend on p < 0.08.

### PTSD, Depressive Symptoms and Alcohol Use

Thirty-nine participants (69.6%) reported an event that fulfilled the trauma criterion A according to the DSM-IV. In 30.4% (*n* = 17) of the cases, the most upsetting event was related to an assault (sexual or physical) by a family member or someone known, in another 16.1% (*n* = 9) it was an aggressive act by a stranger. 30.4% (*n* = 17) of the participants reported an event in the category “any other traumatic event” as being their most upsetting.

The prevalence for current PTSD was high, with 25% of the participants (*n* = 14) fulfilling DSM-IV criteria for the disorder. Table 3 provides an overview on number of trauma symptoms and severity scores.

**TABLE 3:** **PTSD prevalence and trauma symptom severity**.

PTSD diagnosis, *n* (%)	14 (25.0)
Mean PTSD severity score *for participants with PTSD* (*SD*)	19.14 (5.79)
Moderate severity, *n* (%)	8 (57.2)
Moderate to severe severity, *n* (%)	6 (42.9)
Mean number of PTSD symptoms *for participants with PTSD* (*SD*)	12.57 (2.14)
Mean number of impaired areas of psychosocial functioning *for participants with PTSD* (*SD*)	4.93 (1.77)

Mean depression score on the HSCL was 1.55 (SD = 0.43). 26.8% (*n* = 15) of the sample scored above the cut-off score for clinically relevant depression. Applying the available norms for the German general population (Glaesmer et al., 2014), 50% (*n* = 28) of the participants had normal depression scores, 14.3% (*n* = 8) had marginally abnormal scores, 5.4% (*n* = 3) had strongly abnormal scores, and 30.4% (*n* = 17) had extremely abnormal depression scores on the HSCL. Ten participants (18.2%) reported a suicide attempt in the past.

Asked about their drinking habits, 35.7% (*n* = 20) of the sample reported that they did not drink alcohol at all or less than once a month. 14.3% (*n* = 8) of the participants reported drinking two to four times a month and another 14.3% (*n* = 8) stated to drink two to four times a week. Four individuals (7%) reported to drink four or more times a week. The same number of participants had tried to stop drinking at least once in the past 12 months. Six individuals (11% of the sample) reported that a relative, friend or health worker had expressed concern about their drinking. Only one participant reported consumption of any other substance.

### Associations Between Child Abuse and Psychopathology

Predictors of PTSD were established by calculating a linear regression model on the PDS symptom severity score (see Table 4). An analysis of standard residuals was carried out, which showed that the data contained no outliers (Std. Residual Min = –1.73, Std. Residual Max = 2.94). Additional tests and visual inspection of data distribution revealed that the data met the assumptions of collinearity, independent errors, normal distribution of the standardised residuals, homogeneity of variance, and linearity.

**TABLE 4:** **Prediction of PTSD symptom severity: standardized beta coefficients and zero-order correlation coefficients resulting from a linear regression model on PDS symptom severity score**.

**Predictor**	**β**	**Zero-order correlation**
Age	0.08	–0.19
Gender (female)	0.07	–0.11
Exposure to intimate partner violence (yes)	0.08	0.02
Number of traumatic events	0.23	0.47**
Amount of family violence	0.50**	0.65**
Amount of institutional violence	0.05	0.49**

Full model’s adjusted R^2^ = 0.40; F(6.44) = 6.63, p < 0.0001. Zero-order correlation is represented by Spearman’s Rho for continuous predictor variables and point-biserial correlation for dichotomous predictor variables. **Indicate correlations significant on p < 0.01.

The regression analysis showed that the amount of child abuse experiences in the family (family violence) was the only significant predictor for PTSD symptom severity.

**FIGURE 2:**
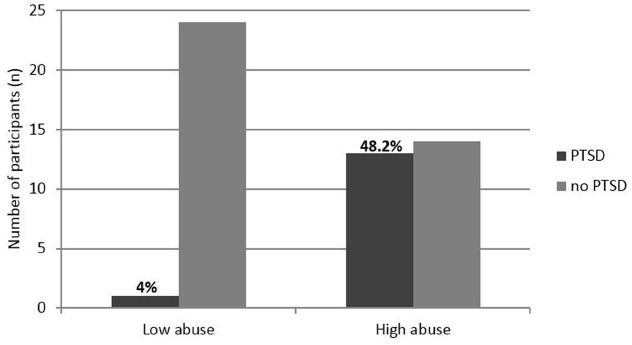
**Frequency (***n***) of PTSD diagnosis for participants in the “high abuse” and “low abuse” group**.

To further investigate the link between the amount of family violence and psychopathology, we subdivided the sample into two groups based on a median split for the total amount of different types of child abuse experiences that had happened in the family (high abuse >4; low abuse ≤4). A Chi-square test was used to verify the difference in percentage of PTSD diagnosis between the two subgroups. Differences on the HSCL depression score were analyzed by the Student’s two-tailed *t*-test for unpaired samples. As illustrated in Figure 2, PTSD prevalence of participants with a high amount of child abuse experiences in their families (“high abuse”) was significantly higher (*χ*^2^ = 12.86; *p* < 0.001) compared to the “low abuse” group.

In addition, the “high abuse” subgroup reported a significantly higher score (*t* = 2.54; *p* < 0.05) on the HSCL depression scale (*M* = 1.69; SD = 0.47) compared to the “low abuse” group (*M* = 1.41; SD = 0.15).

## Discussion

By examining adverse and traumatic life experiences and PTSD symptoms in an unselected sample of adults with ID, the present study revealed high rates of child maltreatment related to both family abuse and victimization in an institutional setting. The prevalence rate for current PTSD was very high with 25% of the participants fulfilling DSM-IV criteria for the disorder. A key finding was that the amount of child abuse in the family emerged as an independent predictor of current PTSD symptom severity, suggesting an enduring impact of early maltreatment experiences on adult mental health in individuals with ID.

The majority of the participants (87.5%) described a least one type of abusive experience occurring in their families, with emotional and physical abuse being almost equally common (76.8 vs. 73.2%). Experience of severe physical violence such as being badly beaten (44.6%) and being beaten hit with an object (23.2%) were frequently reported. These numbers substantially exceed child abuse prevalence rates from the general population, which range from 12 to 14% for physical abuse and 10–15% for emotional abuse and neglect (Finkelhor et al., 2005; Häuser et al., 2011; Iffland et al., 2013). Childhood experience of sexual violence was reported by 12.5% of the individuals in the present study, which is also elevated compared to data from the non-disabled population. This finding is in agreement with prevalence rates for child sexual abuse reported in prior research examining adults and children with (intellectual) disabilities (Sullivan and Knutson, 2000a; Jones et al., 2012). Physical abuse and neglect are reported more frequently in the present study when compared to previous findings in disabled populations; however, a direct comparison is difficult due to differences in the operationalization of “maltreatment” and an often inadequate specification of the disability type (Mikton and Shakespeare, 2014). Nevertheless, based on the results of the present study, specifically that 43% of the participants reported having suffered at least one injury and almost one in five (18%) had needed medical treatment because of the abuse, we can conclude that the level of childhood exposure to family violence was exceptionally high. In addition, it seems that violence was not exclusively directed toward the child with ID as 43% of the participants reported that they had witnessed their mother being beaten and 30% stated that their siblings were also beaten. This finding challenges the view that violence by caretakers is specifically directed toward the disabled child because increased rates of conduct, attention or compliance problems (Larzelere, 2000; Alizadeh et al., 2007) increase parents’ stress and lead to frustrated or angry reactive parenting behavior (Hendricks et al., 2014). Rather, it appears that the level of intrafamilial violence in general is increased when one child is affected by ID as this condition often places greater emotional, economic and social demands on the family as a whole. This concords with the finding that in the presence of adverse circumstances, different risk factors tend to accumulate (Rutter, 1985). Family characteristics such as unemployment, social isolation, financial difficulties and interparental violence have all been associated with a higher risk of child maltreatment (Edleson, 1999; Hamilton-Giachritsis and Browne, 2005).

Based on the present findings, we can assume that violence against people with ID is not only limited to the family context, but also takes place within institutions intended to protect and educate disabled children such as in schools, daycare units or children’s homes. Around 80% of the present sample reported at least one abusive experience in institutions, with physical abuse being the most frequent from of institutional violence (reported by 77%). We are unable to classify the severity of the abuse in the present study as we did not use a questionnaire with validated cut-offs for the presence of abuse. However, the fact that almost 45% of the individuals stated that they had been badly beaten by caretakers in their institutions hints at the alarming magnitude of the problem. Unfortunately, to date there is little valid data available about the frequency of institutional violence committed against children with disability, particularly in relation to non-residential institutions. Based on analysis of historical records of residential facilities, it is clear that a residential placement is a major risk factor for experiencing sexual or physical abuse for disabled children (Sullivan, 2009). In sum, the present retrospective data indicate elevated rated of institutional abuse toward children with disabilities and highlight the need for more thorough research on the prevalence and nature of this phenomenon and its consequences for child and adult mental health.

There were numerous intercorrelations between the various forms of victimization experiences. Physical and emotional child abuse was positively correlated with the amount of institutional violence and the number of general traumatic events, whereas childhood sexual abuse was related to the experience of IPV in adult life. Both results are in agreement with the well-known finding in non-disabled persons that prior exposure to violence constitutes an important risk factor for re-victimization (Widom et al., 2008). 45.8% of the females (compared to 10.7% of men) stated that they had experienced physical violence in an intimate relationship. In addition, more than half of the sample had been physically assaulted by a stranger. These results demonstrate that individuals with ID (and particularly women) are at high risk of experiencing interpersonal violence, not only during childhood but also later in life. This is consistent with the findings of a study exploring the prevalence of violent crimes against persons with disabilities (Rand and Harrell, 2009). It has been suggested that individuals with ID might be especially vulnerable to revictimization because of their impairments in judgment, social skills and communication (Weiss et al., 2011) and due to the fact that they are often socially isolated and therefore less likely to seek help and report assaults to others. The elevated risk for sexual assault in women with ID might also be related to a lack of knowledge about appropriate sexual behavior and inadequate self-protective strategies against abuse (Sobsey, 1997). The present findings point to the need for targeted interventions aimed at both reducing the adverse effects of abuse in children with ID and also promoting effective prevention strategies against further victimization in adulthood.

The consequences of the extensive amount of abusive experiences reported by the present sample are reflected in a high PTSD prevalence (25%) as well as an elevated occurrence of clinically relevant symptoms of depression (27%). It is not possible to compare these findings with others because of the absence of studies investigating prevalence of mental illness such as PTSD in samples of persons with ID. However, outcomes in the present study are similar to the prevalence rates of mental health disorders that have been published for non-disabled adult survivors of child abuse (Widom, 1999; Scott et al., 2012). Our initial assumption of a cumulative effect of different stressors on current PTSD was confirmed only on a correlational level whereas a regression analysis revealed the amount of child abuse in the family to be the only significant independent predictor of PTSD symptom severity. Again, as there are no comparable studies with individuals with ID, we can only discuss this finding based on the evidence that has been described for the general population or non-disabled samples. The relationship between child abuse and adult PTSD is well established (Widom, 1999; Stovall-McClough and Cloitre, 2006; Heim and Nemeroff, 2015) and a number of factors have been proposed to account for this association. For instance, child abuse may alter attachment patterns as well as neurobiological developmental processes (Watts-English et al., 2006) including interactions with genetic factors (Mehta and Binder, 2012) thereby enhancing the risk of subsequent development of PTSD. The presence of ID might further increase this vulnerability since previous longitudinal studies have revealed that a lower IQ in young children heightened their risk for PTSD in response to subsequent traumatic events (Breslau et al., 2006; Koenen et al., 2007).

All but one individual with a PTSD diagnosis had experienced a high incidence of abuse, thereby validating the importance of child abuse in subsequent development of PTSD during adulthood as well as in symptom severity. A number of studies have shown that childhood maltreatment is a key determinant for the development of depression (Anda et al., 2002; Norman et al., 2012). Similarly in the present study, individuals who had experienced more severe levels of child abuse also reported higher levels of depressive symptoms. The extent of child abuse in the family was related to both a higher number of adverse and traumatic experiences as well as to adult mental health problems, highlighting how essential family factors and particularly parenting practices are for the development of children with ID. In fact, whilst experiences of institutional violence, IPV, and aggression by strangers were frequent in our sample, none of these adversities was associated with current PTSD symptom severity if the amount of childhood family violence was taken into account. These findings underscore the urgent need to support families with children with ID and to promote supportive and non-violent ways of parenting in order to reduce the risk for later re-victimization and to encourage healthy development in intellectually disabled individuals.

### Limitations

The current study contributes new insight into the association between child abuse and adult mental health in individuals with ID. However, several limitations have to be acknowledged. Firstly, the sample size is small and participants were not tested with a formal IQ test to validate the diagnosis of ID. In addition, the sample was recruited through a welfare center that specialized in the care of individuals with mostly mild and moderate forms of ID. Thus, findings cannot be generalized to the entire population of people with ID. Despite efforts to obtain an unselected sample within the welfare center, a number of individuals refused to take part. This might have biased our results. However, the fact that the most common reasons for refusal were fear of talking to strangers or being too burdened might give rise to the assumption that disproportionately many individuals with a particularly high stress load were among the people who did not take part in the study. We might therefore assume that the present findings were biased more likely toward an underestimation the frequency of abusive and stressful experiences and current mental health problems. In addition, we cannot rule out that the presence of the social worker during the interview might have influenced the participants’ answers.

Furthermore, our cross-sectional design does not allow for verification of hypothesized causal relationships. In fact, the potential for reverse causation, i.e., disability arises as a result of child abuse (Govindshenoy and Spencer, 2007), cannot be ruled out, even though many participants reported that their disabilities occurred from birth or soon afterward. Longitudinal studies are necessary in order to clarify whether (intellectual) disabilities are a risk factor or rather an outcome of child abuse, or both. Longitudinal designs would have the additional advantage of avoiding potential report bias in adults regarding retrospective events.

One of the most important limitations of the present study is the fact that, we did not assess peer victimization and bullying experiences. The evidence that people with ID have an increased risk of being victimized by their peers (Jones et al., 2012) together with the recent knowledge about long-term consequences of peer bullying for adult mental health (Lereya et al., 2015) may give rise to the assumption that such experiences might have an additional impact in the present sample. Future research should address the frequency of peer victimization and related consequences for the development of children and youth with ID.

## Conclusion

Child maltreatment seems to play a decisive role for the development of mental health problems and exposure to further trauma amongst intellectually disabled individuals. The current study indicates high prevalence of child maltreatment, which is strongly associated with other forms of victimization, such as institutional abuse or IPV, and is an independent risk factor for the severity of adult PTSD, a disorder diagnosed in a quarter of the participants. In order to reduce the frequent victimization of intellectually disabled people across different contexts, intervention and prevention programs should start at the family level. Parent training programs should be tailored to the specific needs of families with an intellectually disabled child and should teach parents non-violent and supportive ways of educating their children. In addition, targeted preventive measures should be designed to empower children with ID to recognize abuse and grooming and to effectively seek help. Finally, given the high PTSD rate in populations with intellectual impairments, further research is required to see whether current therapeutic approaches with proven efficacy in non-disabled populations are also effective in this group, or whether adaptations to current treatments are required.

## Author Contributions

CC conceived the study, developed the design, participated in and supervised data acquisition, performed the statistical analysis and drafted the manuscript. IS carried out clinical interviews, entered the data, and revised the manuscript critically for important intellectual content. Both authors gave their final approval of the current version of the manuscript. The authors agree to be accountable for all aspects of the work in ensuring that questions related to the accuracy or integrity of any part of the work are appropriately investigated and resolved.

### Conflict of Interest Statement

The authors declare that the research was conducted in the absence of any commercial or financial relationships that could be construed as a potential conflict of interest.
